# Editorial commentary on the special issue of cancer research

**DOI:** 10.7555/JBR.38.20240800

**Published:** 2024-07

**Authors:** Editorial Board

Cancer research is at the forefront of medical science, aimed at clarifying the pathogenesis of carcinogenesis as well as prevention, diagnosis, and treatment of cancers. Significant advances have been made in precision medicine, immunotherapy, and cell therapy, among others. In this special issue, we publish nine articles covering various topics, such as potential application of miRNAs in bladder cancer diagnosis and treatment, molecular mechanisms of carcinogenesis, plant-derived antitumor drugs, glioma angiogenesis inhibition, acute myeloid leukemia (AML) prognostic markers, therapeutic cancer vaccines, and genetic risk factors for gastric and prostate cancer.

Wang *et al*^[1]^ conducted a comprehensive analysis of miRNA research in bladder cancer, highlighting the potential of miRNAs in the diagnosis, treatment, and prognosis of bladder cancer, as well as the challenges in potential clinical applications.

Gu *et al*^[2]^ summarized the research progress in liquid-liquid phase separation, its role in transcriptional regulation and carcinogenesis, and the latest research strategies for the targeted phase separation therapy of cancers, providing new directions for the development of novel cancer treatment.

Min *et al*^[3]^ reported that moroidin, a plant-derived cyclopeptide, suppressed the formation of vasculogenic mimicry in glioblastoma cells by inhibiting β-catenin activation, and thereby modulating epithelial-mesenchymal transition, offering a potential therapeutic strategy to overcome resistance to the reported angiogenesis inhibitors currently used in the treatment of glioblastoma.

Chen *et al*^[4]^ demonstrated that p53 modulated the super-enhancer landscape, thereby affecting genes critical for lung cancer development, and identified KLF4 as a p53-dependent super-enhancer with tumor suppressor properties, providing new insights into the role of p53 in lung carcinogenesis.

Guo *et al*^[5]^ investigated the associations of genetic variants in *C1GALT1* (*i.e.*, rs35999583G>C) with gastric cancer risk and examined their immunoregulatory effects on the tumor microenvironment, offering a promising predictor of gastric cancer risk and immune status.

Cheng *et al*^[6]^ identified a genetic variant in *circHIBADH* (*i.e.*, rs11973492T>C) that was associated with an increased prostate cancer risk, and further investigated its role in RNA splicing regulation and its potential as a therapeutic target.

Qian *et al*^[7]^ reported that shikonin and *Mycobacterium smegmatis* synergistically induced immunogenic cell death, presenting a promising *in situ* tumor vaccine approach to enhance systemic antitumor immunity.

Hafiz *et al*^[8]^ demonstrated the novel role of timosaponin AⅢ as a constitutive androstane receptor (CAR) activator that affected drug metabolism, highlighting its potential in cancer therapy and drug-drug interaction management.

Tang *et al*^[9]^ developed a prognostic model including nine cell surface markers for risk stratification of AML patients based on a multi-model analysis, validated its predictive value as an independent prognostic factor for survival in AML patients, and demonstrated the model's potential for guiding drug therapy.

We hope the readers find this special issue interesting and intriguing.
